# Endogenous AJAP1 associates with the cytoskeleton and attenuates angiogenesis in endothelial cells

**DOI:** 10.1242/bio.022335

**Published:** 2017-05-08

**Authors:** Katharina Hötte, Isabell Smyrek, Anna Starzinski-Powitz, Ernst H. K. Stelzer

**Affiliations:** 1 Physical Biology/Physikalische Biologie (IZN, FB 15), Buchmann Institute for Molecular Life Sciences (BMLS), Cluster of Excellence Frankfurt – Macromolecular Complexes (CEF – MC), Goethe Universität – Frankfurt am Main (Campus Riedberg), Max-von-Laue-Straße 15, Frankfurt am Main D-60438, Germany; 2 Institute of Cell Biology and Neuroscience, Department of Molecular Cell Biology and Human Genetics, Goethe Universität – Frankfurt am Main (Campus Riedberg), Max-von-Laue-Straße 13, Frankfurt am Main D-60438, Germany

**Keywords:** AJAP1, Shrew-1, Angiogenesis, Cell migration, Microtubules, Endothelial cells, Cytoskeleton

## Abstract

The adherens junction associated protein 1 (AJAP1, aka shrew-1) is presumably a type-I transmembrane protein localizing and interacting with the E-cadherin-catenin complex. In various tumors, AJAP1 expression is reduced or lost, including hepatocellular and esophageal squamous cell carcinoma, and glial-derived tumors. The aberrant expression of AJAP1 is associated with alterations in cell migration, invasion, increased tumor growth, and tumor vascularization, suggesting AJAP1 as a putative tumor suppressor. We show that AJAP1 attenuates sprouting angiogenesis by reducing endothelial migration and invasion capacities. Further, we show for the first time that endogenous AJAP1 is associated with the microtubule cytoskeleton. This linkage is independent from cell confluency and stable during angiogenic sprouting *in vitro*. Our work suggests that AJAP1 is a putative negative regulator of angiogenesis, reducing cell migration and invasion by interfering with the microtubule network. Based on our results and those of other authors, we suggest AJAP1 as a novel tumor suppressor and diagnostic marker.

## Abstract (precis)

**Summary:** Depletion of AJAP1 in endothelial cells can increase angiogenic-like sprouting and cell migration. In addition, AJAP1 associates with microtubules in these cells.

## INTRODUCTION

The adherens junction-associated protein 1 (AJAP1), also known as shrew-1, has been suggested as a type-I transmembrane protein. Originally, it has been identified in an invasive cell line from endometriosis lesions (Bharti et al., 2004). In polarized epithelial cells, AJAP1 localizes and interacts with β-catenin in the E-cadherin-catenin complex and is found in cell-cell contacts in the human mammary gland, the uterus, breast carcinoma cells (Gross et al., 2009; Jakob et al., 2006) and in glioblastoma cell lines (Han et al., 2014). Recently, we reported the identification of two other AJAP1 protein isoforms (isoform 2 and 3), which result from an altered exon usage producing different AJAP1 transcript variants. These isoforms differ in length and show different expression patterns between tissues and during development. In mammary tissue sections of virgin mice, AJAP1 is localized in the cytoplasm, while during alveologenesis it translocates to the nucleus. Isoform 2, like isoform 1, comprises an extracellular and intracellular domain, whereas the first eleven amino acids are truncated. Isoform 3 consists of only 120 amino acids, and the extracellular domain is truncated (Klemmt et al., 2016).

It has been shown that the expression of AJAP1 is dysregulated in various cancer types including glioma (Lin et al., 2012; McDonald et al., 2006), hepatocellular carcinoma (HCC) (Ezaka et al., 2015) and esophageal squamous cell carcinoma (ESCC) (Tanaka et al., 2015).

In these contexts AJAP1 influences cell migration, invasion and proliferation, though its modulatory effect appears to depend on the cellular context. In breast cancer cells, AJAP1 has been shown to accelerate the process of wound closure, while the downregulation of AJAP1 reduces the migratory capacity of cells (Gross et al., 2009). Furthermore, in non-polarized cells AJAP1 promotes migration and invasion by directly interacting with the transmembrane glycoprotein and extracellular matrix metalloproteinase (MMP) inducer CD147 (Schreiner et al., 2007).

AJAP1 has been studied in glioblastoma, which is one of the most common and most malignant primary tumors in the central nervous system associated with poor patient survival rates (Smith and Jenkins, 2000). In humans, the *AJAP1* gene is localized on the mutational hotspot of the 1p36 locus. Deletion or epigenetic silencing, caused by hyper-methylation of the proximal promoter as shown in glioblastoma tumors, leads to loss of *AJAP1* and is associated with cancer development (Ernst et al., 2009; McDonald et al., 2006; Lin et al., 2012). In this context, glioma cells stably overexpressing AJAP1 show a reduced migratory capacity compared to wild-type cells suggesting that AJAP1 actually has an inhibiting effect on cell migration (McDonald et al., 2006). Overexpression of AJAP1 in oligodendroglioma cell lines indicates that AJAP1 localizes at the adherens junctions, where it could interact with β-catenin (Chen et al., 2014; Zeng et al., 2014). In surgical sections of diffuse astrocytoma, AJAP1 localizes at the cell membrane, whereas in oligodendroglioma sections AJAP1 is not detectable at all (Zeng et al., 2014).

AJAP1 suppresses cell proliferation, migration and invasion and alters cytoskeletal reorganization in glioblastoma *in vitro* and *in vivo* by as yet unknown mechanisms (Han et al., 2014). In HCC and ESCC, the expression of *AJAP1* was also found to be reduced due to promotor hyper-methylation and loss of copy number, suggesting that AJAP1 acts as a tumor suppressor (Ezaka et al., 2015; Tanaka et al., 2015). The tumor size and vascular invasion inversely correlate with AJAP1 mRNA levels in HCC (Ezaka et al., 2015).

Angiogenesis is involved in physiologic processes in different stages of development, adulthood (female reproductive cycle and wound healing), as well as in pathologic processes such as tumor growth and formation of metastases (Carmeliet et al., 2009; Drake, 2003; Andres and Djonov, 2010). During tumor angiogenesis, *de novo* blood vessel formation is initiated by the unbalanced secretion of vascular endothelial growth factor A (VEGFA) by tumors and involves many of the same processes as those involved in physiological angiogenesis (reviewed in Nagy et al., 2009). These processes include the remodeling of the basement membrane and the extracellular matrix (ECM) aided by MMPs, followed by endothelial cell proliferation, migration and invasion towards an angiogenic stimulus. Finally, new blood vessels are formed. Tumor angiogenesis generates abnormal blood vessels, which are often found to be unevenly distributed and irregularly branched (reviewed in Nussenbaum and Herman, 2010). Tumor angiogenesis is a hallmark for tumor progression and has become a well-investigated target for cancer treatment.

Our motivation was to investigate the role of endogenous AJAP1 in endothelial cells, in particular the effect of AJAP1 on cell migration and sprouting angiogenesis.

We hypothesized that AJAP1 is important in sprouting angiogenesis and thereby potentially influences the vascularization of tumors. Our data show that downregulation of AJAP1 leads to an increase in the cumulative sprout length during sprouting angiogenesis. AJAP1 downregulation enhanced the cell migration of human primary endothelial cells. By investigating the endogenous protein localization in endothelial cells, we found that AJAP1 co-localized with the microtubule cytoskeleton.

For the first time, we show that endogenous AJAP1 associates with the microtubule cytoskeleton and that AJAP1 influences sprouting angiogenesis. This accompanies the negative impact on cell migration in endothelial cells. Thus, our research underpins the importance of AJAP1 in cell migration and invasion during sprouting angiogenesis, probably due to its interaction with the microtubule cytoskeleton. Our research, together with previous studies, shows that tumor growth behavior and vascularization are strongly influenced by AJAP1, hence suggesting AJAP1 as a tumor marker for the malignancy of different cancer types.

## RESULTS

### AJAP1 knockdown induces angiogenic sprouting *in vitro*

AJAP1 influences cell migration and invasion in epithelial cells by modulating cell-surface levels of E-cadherin (Gross et al., 2009). However, in highly invasive oligodendroglioma cells, AJAP1 is epigenetically down-regulated. Restoration by overexpression of AJAP1 in these cells results in a reduction of cell migration and invasion (Han et al., 2014; Cogdell et al., 2011; McDonald et al., 2006). Increased tumor vascularization and invasion inversely correlate with the mRNA levels of AJAP1, indicating that AJAP1 is important for cell and tissue integrity and that loss of AJAP1 promotes tumor progression.

**Fig. 1.**
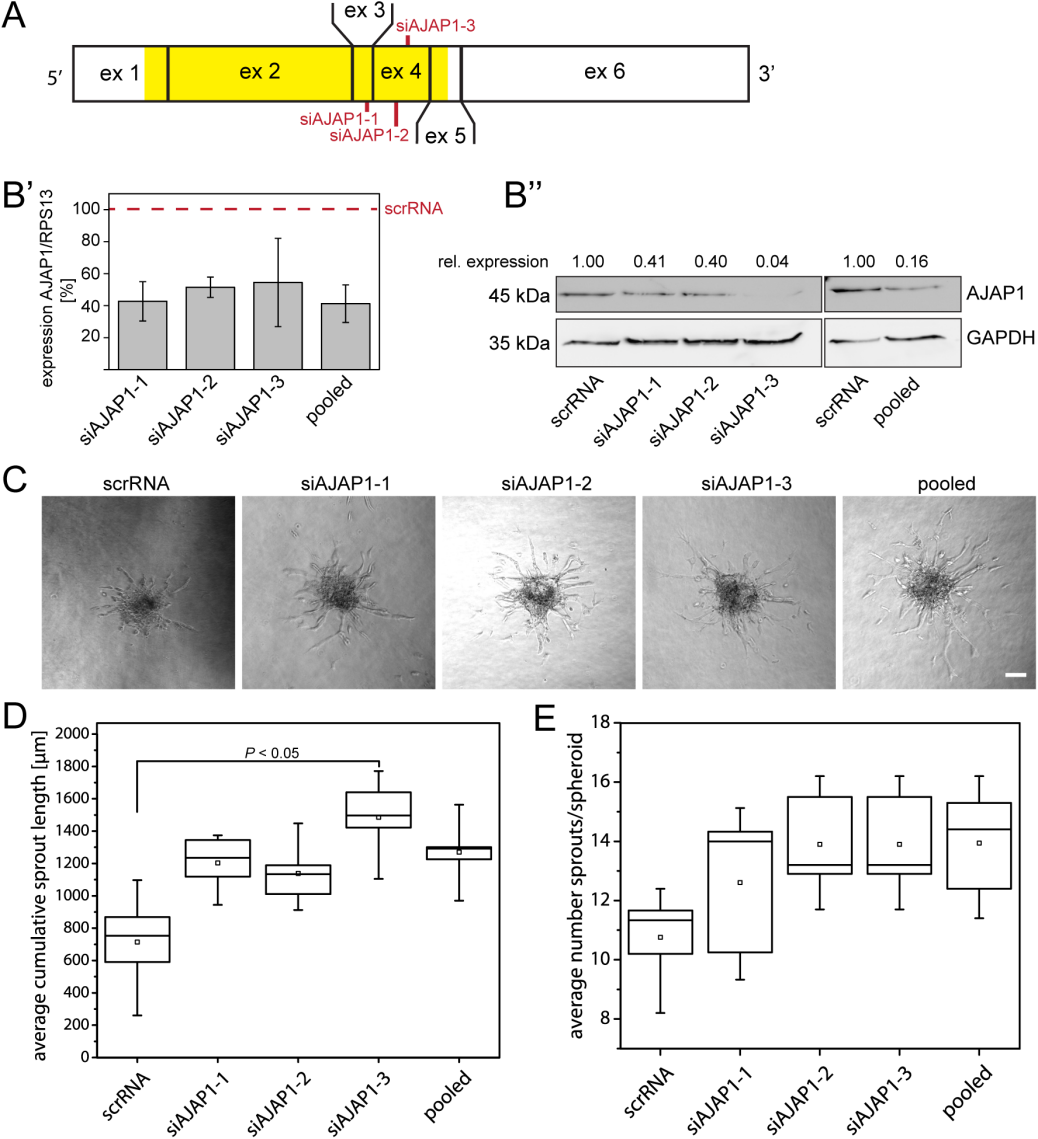
**AJAP1 downregulation increases the sprout formation in the HUVEC sprouting assay.** (A) Schematic overview of the siRNA binding sites for AJAP1 mRNA. Three different siRNAs targeting two distinct exons on human AJAP1 mRNA were transfected in HUVECs. Red marks indicate the respective siRNA binding site. Yellow indicates the protein coding region on AJAP1 mRNA. Ex, exon. (B′) The knockdown efficiency of each experiment was determined separately by measuring the relative AJAP1 mRNA expression compared to RPS13 by qRT-PCR. The expression levels were normalized to relative mRNA expression of cells transfected with scrRNA. The plot shows mean±s.d. of the five performed experiments. (B″) AJAP1 downregulation on protein level was confirmed by western blot. The relative amount of AJAP1 to GAPDH is shown. A representative example is shown. (C) Representative samples of sprouting spheroids are shown 24 h after embedment into collagen I. Microscope: Axiovert 40 CFL; objective lens: A-Plan 10×/0.25Ph; scale bar: 50 µm. (D,E). The average cumulative sprout length and the average number of sprouts per spheroid was quantified and is presented in a box and whiskers plot. The box contains 50% of the data points, the middle line of the box is the median and the square is the arithmetic mean. Whiskers represent minimum/maximum values. Per condition, five independent experiments with ten replicates were performed. Statistics was performed using the *t*-test with post hoc Bonferroni correction for multiple comparison between all conditions.

We investigated the role of AJAP1 in endothelial cells, in particular during angiogenic sprouting, by examining the effect of siRNA-mediated AJAP1 knockdown on the sprouting activity in a spheroid-based angiogenesis assay in human umbilical vein endothelial cells (HUVECs). Three different siRNAs were used which target either exon 3 (siAJAP1-1) or exon 4 (siAJAP-2 and -3) and are known to be expressed in all known AJAP1 isoforms (Fig. 1A). All of these, singular as well as pooled, were able to downregulate AJAP1 expression on the mRNA and on the protein level (Fig. 1B′,B″). For all knockdown experiments, including the pooled siRNAs, we used 12.5 µM of siRNA in total. The remaining expression of AJAP1 mRNA showed variations but was similar between all knockdown conditions. Interestingly, siAJAP1-3 was found to have the strongest effect on the resulting protein expression.

The effect of AJAP1 downregulation on sprouting angiogenesis was evaluated by measuring the cumulative sprout length, which includes the number and length of the main sprouts and the number and length of the branches. We found that AJAP1 knockdown enhanced sprout growth 24 h after embedment of HUVEC spheroids into collagen gels. When cells were transfected with siAJAP1-3 the average cumulative sprout length was significantly increased by 108.2% (Fig. 1D). The average number of sprouts per spheroid was slightly but not significantly increased when AJAP1 was downregulated (Fig. 1E).

**Fig. 2.**
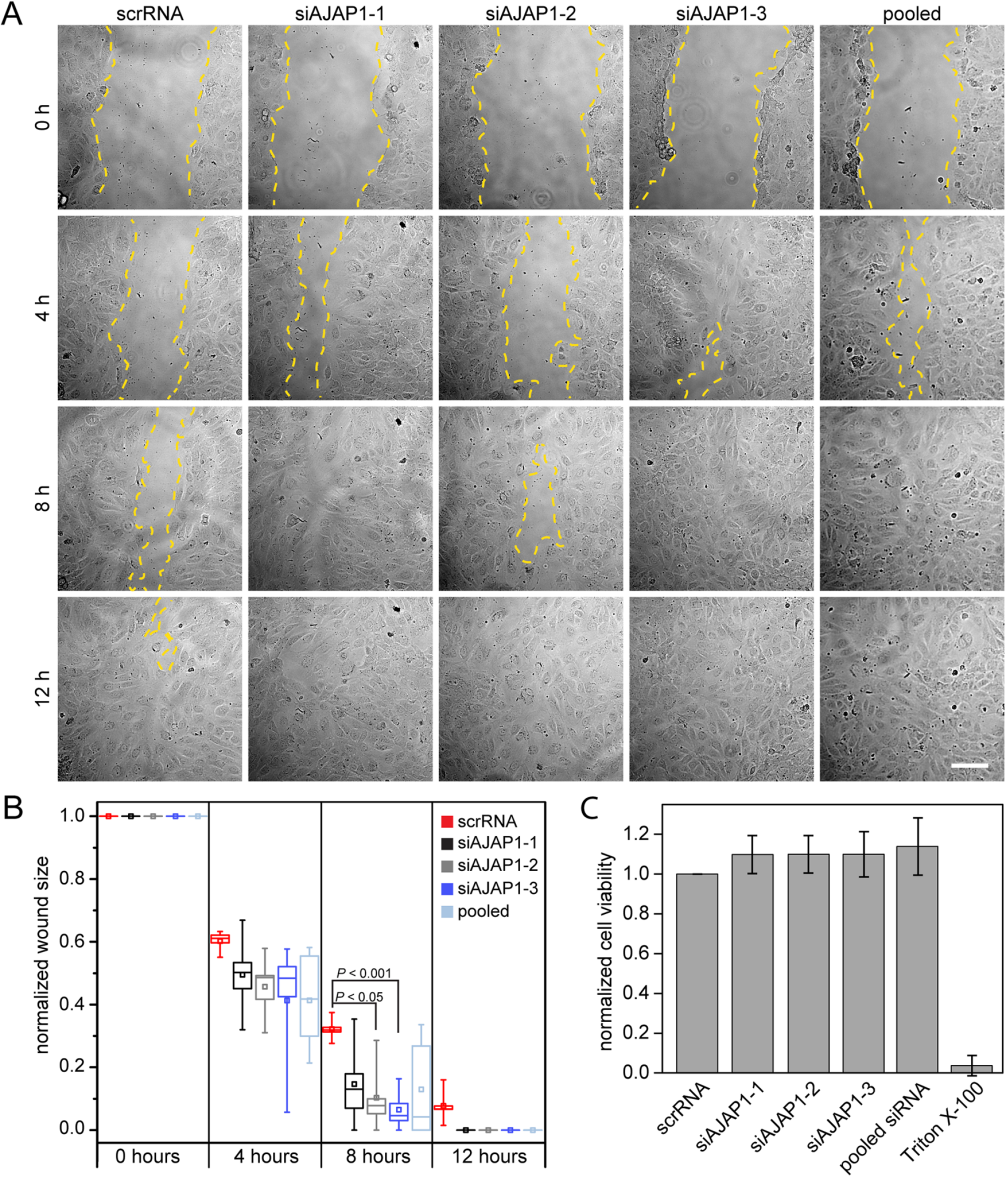
**AJAP1 knockdown induces wound closure in HUVECs.** (A) Migration of scrRNA, siAJAP1-1, siAJAP1-2, siAJAP1-3 and pooled siRNA transfected HUVECs in an *in vitro* wound healing assay at different time points after wounding. The yellow dashed line indicates the migrating front. Microscope: Zeiss Axio Observer.Z1; objective lens: Fluar 10×/0.5; scale bar: 100 µm. (B) The normalized wound size is plotted for each time point and each condition. The wound size decreases faster over time when AJAP1 is down regulated. Five independent experiments were performed per condition. Data is normalized to the scratch size at time point 0 for each condition. Only samples with a comparable wound area size were chosen for the analysis. The box contains 50% of the data points, the middle line of the box is the median and the square is the arithmetic mean. Whiskers represent minimum/maximum values. Statistics were performed using the *t*-test with the Bonferroni correction. (C) HUVECs were transfected with scrRNA or siAJAP1. Cell viability was measured by a MTS assay and is expressed as a percentage of scrRNA transfected cells. Mean±s.d. *n*=3.

The spheroid-based sprouting assay represents several aspects of angiogenesis. The formation of new sprouts requires cells to remodel the ECM in order to allow migration towards the pro-angiogenic stimulus. To further characterize AJAP1 in the endothelial context, we performed a scratched wound healing assay to monitor the effect on basal cell migration *in vitro*. HUVECs were transfected with siAJAP1 or scrRNA 48 h before the wound was applied to the confluent grown cells. The migration of HUVECs with a reduced AJAP1 expression was faster, resulting in a remaining average wound size of 14.7% (siAJAP1-1), 10.3% (siAJAP1-2, *P*<0.05), 6.5% (siAJAP1-3, *P*<0.001) and 12.9% (pooled siRNA) compared to the control, which had a remaining wound area of 32.2% 8 h following wound setting (Fig. 2A,B). To estimate whether the difference in wound closure was an effect of altered cell proliferation, we performed a MTS assay with HUVECs with knockdown for AJAP1 or control. The cell viability of HUVECs with AJAP1 knockdown was not significantly higher compared to the control (Fig. 2C).

This data suggested that AJAP1 is either directly or indirectly involved in endothelial sprout formation. When AJAP1 was silenced, the overall sprout length was significantly increased. Supported by the scratched wound healing assay, these results showed that the knockdown of AJAP1 had a positive influence on cell migration in human endothelial cells, which was not caused by increased cell viability, suggesting that AJAP1 influences the migration process itself. Moreover, the three-dimensional sprouting assay demonstrated rather cell invasion into collagen gels than only cell migration, suggesting a role of AJAP1 in this process.

### AJAP1 localizes at microtubules in endothelial cells

**Fig. 3.**
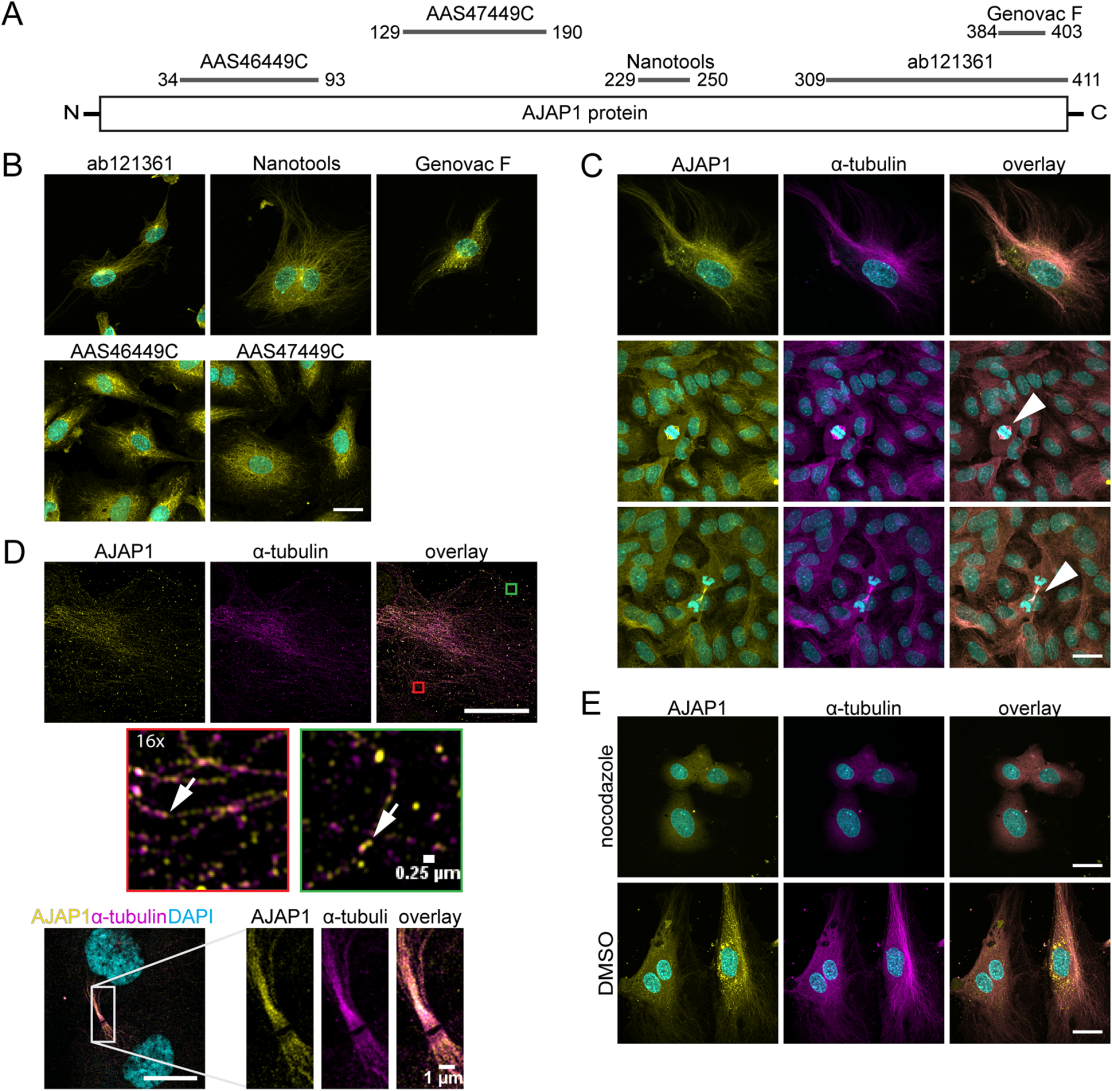
**AJAP1 co-localizes with microtubules in HUVECs.** (A) Schematic overview of the epitope binding sites for antibodies used to detect AJAP1 in HUVECs. The antibody recognition sites are mapped to the polypeptide chain. The numbers indicate the amino acid positions on the AJAP1 polypeptide chain. (B) Detection of AJAP1 (yellow) using antibodies with different epitope recognition sites shows fibrillar structures in HUVECs. Cell nuclei were counterstained with DAPI (cyan). Scale bar: 25 µm. (C) Immunostaining of AJAP1 (Genovac antibody, yellow) and α-tubulin (magenta) shows AJAP1's association with microtubules in HUVECs. The microtubule association is independent from the confluence of the culture. HUVECs were grown until confluence and fixed after seven days. AJAP1 is additionally localized to microtubules contributing to the spindle apparatus during cell division (arrowhead). Further, numerous AJAP1-positive puncta are localized in the perinuclear region. Cell nuclei were counterstained with DAPI (cyan). Microscope: Zeiss LSM 780; objective lens: Plan-Apochromat 63×/1.40 Oil DIC M27; scale bar: 25 µm. (D) Structured illumination microscopy (SIM) gives a detailed view of the co-localization of AJAP1 and microtubules in HUVECs. AJAP1 localizes in close proximity to the tubular network lining the fibers (arrows). During mitosis and cell division, AJAP1 co-localizes with the spindle apparatus. Microscope: Zeiss Elyra; objective lens: Plan-Apochromat 63×/1.4 Oil DIC M27; scale bar: 25 µm (upper panel) and 10 µm (lower panel). (E) The association of AJAP1 with microtubules in HUVECs is lost upon microtubule destruction. Treatment with 12.5 µM nocodazole for 24 h shows destruction of the microtubule network and loss of AJAP1 tubular localization. For a negative control, HUVECs are treated with DMSO for 24 h. Cell nuclei were counterstained with DAPI (cyan). Microscope: Zeiss LSM 780; objective lens: 63×/1.40 oil; scale bar: 25 µm.

Initially, it had been shown that AJAP1 localizes at the plasma membrane in the adhesion complex of epithelial cells, where it binds to the E-cadherin-catenin complex (Bharti et al., 2004). However, there is increasing evidence that the expression of AJAP1 is not restricted to the adherens junction, but depends on the developmental stage and tissue type (Klemmt et al., 2016). To assess the molecular function of AJAP1 underlying angiogenic sprouting, we investigated the cellular localization of endogenous AJAP1 in endothelial cells. Therefore, we performed an immunofluorescence staining of HUVECs grown on glass cover slips. We used five antibodies targeting different epitopes on the AJAP1 polypeptide (Fig. 3A). We expected AJAP1 to localize at the cell junctions; however, surprisingly, we saw a fibrillar signal for endogenous AJAP1 in the cytoplasm of these cells but not at the adhesion sites (Fig. 3A; Fig. S1A). All anti-AJAP1 antibodies showed a fibrillar signal in HUVECs. In addition, the Genovac clone F anti-AJAP1 antibody also recognized puncta in the cytoplasm of the cells (Fig. 3B). The structure of the fibrillar signal suggested that AJAP1 was attached to cytoskeletal elements. Co-staining for cytoskeletal components revealed that AJAP1 co-localized with the microtubule cytoskeleton in HUVECs (Fig. 3C) but not with the actin filaments (Fig. S1A). Since this result did not conform with previous studies (Bharti et al., 2004; Gross et al., 2009), we hypothesized that AJAP1 localization in HUVECs depends on the cell density when cell-cell junctions are fully formed. Therefore, we performed an immunofluorescence staining on HUVECs grown until confluence. In fully confluent cells, endogenous AJAP1 remained localized at the microtubules (Fig. 3C). Interestingly, during mitosis, AJAP1 was also detectable at the spindle apparatus (Fig. 3C, arrowhead). In addition, we used structured illumination microscopy (SIM) to verify the association of AJAP1 to the microtubule cytoskeleton with high resolution and to ensure that the co-localization was not an artifact induced by cross-reactivity of the antibodies. We also recorded multi-spectral beads to correct for the spatial misalignment between the spectrally distinct channels (Fig. S1B). Higher resolution of the HUVEC cytoskeleton revealed that the fluorescence signals for AJAP1 and α-tubulin appeared in spots, which were not overlapping completely but rather in close proximity (Fig. 3D). We further applied this technique to dividing cells to obtain more information about the co-localization of AJAP1 and α-tubulin at the spindle apparatus. This result indicated that the strong co-localization between AJAP1 and the microtubules was not an artifact. It further emphasizes that AJAP1 is either directly or indirectly bound to microtubules.

To further verify the association of AJAP1 to the microtubules, we treated HUVECs with 12.5 µM nocodazole to interfere with the polymerization of microtubules. Nocodazole treatment for 24 h showed that the microtubule cytoskeleton was dissociated concomitant with a loss of the fibrillar localization of AJAP1. Diffuse signals for both α-tubulin and AJAP1 were detected in nocodazole-treated HUVECs (Fig. 3E; Fig. S1C). In addition, we examined the appearance of the microtubule cytoskeleton in combination with AJAP1 downregulation. This experiment further aimed to verify the specificity of the anti-AJAP1 antibodies by immunostaining against AJAP1. Following AJAP1 knockdown with pooled siRNAs, we found that AJAP1 itself was not detectable. Under these conditions, the microtubule cytoskeleton was detectable and did not show obvious alterations (Fig. S1D).

The previous literature reports a localization of AJAP1 at the cell adhesion sites in epithelial cells (Gross et al., 2009; Bharti et al., 2004), which opposes our results obtained from endothelial cells. For clarification, we immunostained MCF-7 wild-type and overexpressing cells using the anti-AJAP1 antibodies from Genovac and Nanotools, respectively. We found that the overexpression of AJAP1:EGFP, which reportedly localized to the cell circumference (Gross et al., 2009; Bharti et al., 2004), was not detected by the Nanotools antibody; instead, we found a fibrillar staining pattern. In MCF-7 wild-type cells, a co-stain for α-tubulin revealed that the fibrillar stain obtained with the Genovac anti-AJAP1 antibody co-localized with the microtubule cytoskeleton (Fig. S1E).

These results demonstrated a microtubule association of AJAP1 in endothelial as well as in epithelial cells. In HUVECs, the localization was independent of cell density. Additionally, microtubule association remained stable during mitosis, when AJAP1 was found at the spindle apparatus. Further, the integrity of the microtubule cytoskeleton was not altered upon downregulation of AJAP1.

### AJAP1 steadily associates with microtubules in three-dimensional cell context

**Fig. 4.**
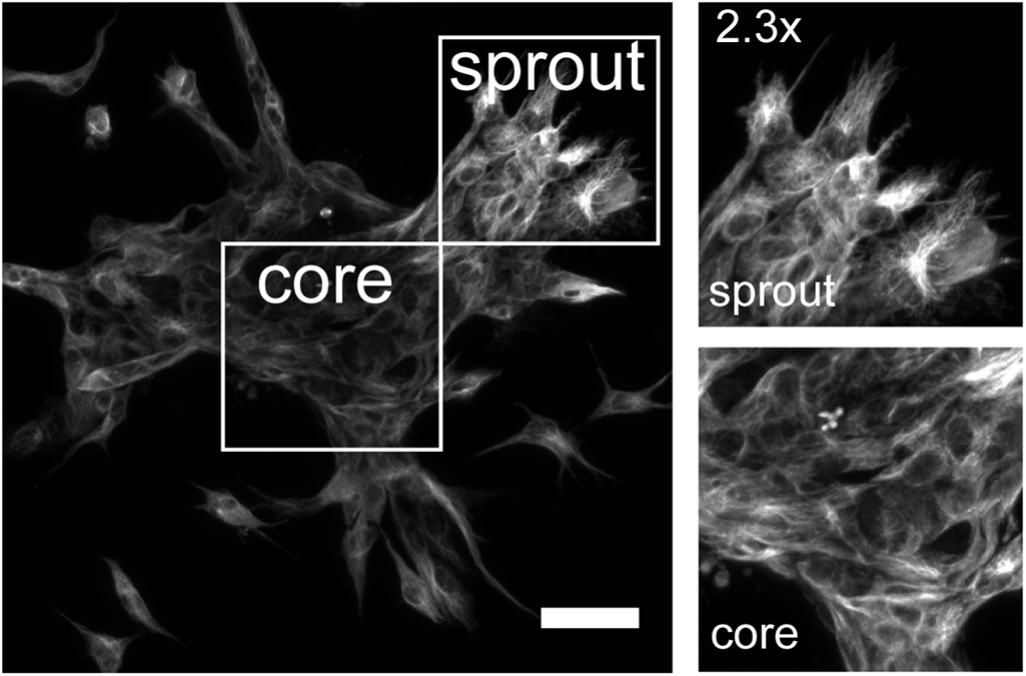
**AJAP1 remains localized in fibrillar structures during HUVEC spheroid sprouting.** The maximum Z-projection shows an immunofluorescence staining of a sprouting spheroid with the localization of AJAP1 (antibody: Abcam). Focusing on the core and the sprouting region, AJAP1 always appears in fibrillar structures indicating that its localization does not alter in polarizing and migrating cells. Microscope: LSM 780; objective lens: LD EC Epiplan-Neofluar 20×/0.22, spacing: 1.58 µm; scale bar: 25 µm.

Next, we addressed the question of whether the observations in our two-dimensional cell culture experiments concerning the intracellular localization of AJAP1 remain consistent in a three-dimensional cell context. In particular, during the spheroid-based sprouting assay cytoskeletal remodeling could induce an altered localization of AJAP1. We assessed this by immunofluorescence staining of the active endothelial cell sprouting process at the end-point of the spheroid-based sprouting experiment. We found that AJAP1 appeared in fibrillar structures in sprouting spheroids. At the leading edge of the sprouts, tip cells were found to be polarized and their microtubules were arranged along the direction of migration. These migrating cells, as well as the cells in the core of the spheroid, showed a fibrillary staining which is similar to the AJAP1 staining of two-dimensional cell cultures (Fig. 4). This indicated that the association of AJAP1 with microtubules was persistent in migrating endothelial cells.

## DISCUSSION

Pathogenesis and survival rate of tumor diseases such as glioblastoma and esophageal cancer have been associated with altered expression of AJAP1, thereby making it an interesting molecular diagnostic marker in cancer therapy (Han et al., 2014; Lin et al., 2012; Tanaka et al., 2015). AJAP1 has been reported to be involved in important cellular processes such as cell migration and invasion by modulating adherens junctions and remodeling of the ECM and the cytoskeleton (Gross et al., 2009; Schreiner et al., 2007; Han et al., 2014).

The influence of AJAP1 on cell migration and invasion has been observed previously in glial-derived tumors (Han et al., 2014; McDonald et al., 2006) and human mammary carcinoma (MCF-7) cells (Gross et al., 2009). Paradoxically, overexpression of AJAP1 in MCF-7 cells increases cell migration and loss of AJAP1 decreases the migration behavior (Gross et al., 2009), whereas in highly invasive glioblastoma tumors and glioblastoma cell lines, AJAP1 expression is lost by methylation of the *AJAP1* promotor. Further, AJAP1 overexpression in glioblastoma cell lines decreases cell migration (Lin et al., 2012; McDonald et al., 2006); our findings in HUVECs are concordant with the studies on glioblastoma. We showed that AJAP1 knockdown enhanced the migratory and invasive behavior of primary endothelial cells in a wound healing and sprouting angiogenesis assay.

Our results demonstrate that a knockdown of AJAP1 influences angiogenic sprouting by significantly increasing the cumulative sprout length. The cumulative sprout length is influenced by different factors: the number and the length of sprouts, and the number and the length of the branches. We tested whether the increase in the cumulative sprout length is due to an increase of the number of sprouts and found that the number of sprouts was increased upon AJAP1 downregulation, but did not show a significant difference. This suggests that the number and the length of branches is increased. However, our measurements do not include the number and length of individual branches, thus we cannot confirm an effect on the number and the length of the branches upon AJAP1 downregulation. This suggests that AJAP1 has an attenuating effect on sprouting angiogenesis. Our results support previous studies, which indicate a significant correlation between reduced AJAP1 mRNA levels and an increased tumor vascularization in patients with hepatocellular carcinoma (Ezaka et al., 2015). Yet, it is not known whether the expression of AJAP1 is altered in the tumor cells and/or the vascular system, or whether the communication between cell types is involved. Vascularization requires a magnitude of cellular processes to be regulated under physiological and pathological conditions, such as cell migration towards an angiogenic stimulus, turnover of cell-cell and cell-matrix contacts, and remodeling of the extracellular matrix (Davis and Senger, 2005; Nishida et al., 2006). Our data suggests that AJAP1 interferes with the process of cell migration, shown by an accelerated wound closure after AJAP1 knockdown.

To understand the role of AJAP1 in cell migration, we examined its cellular localization in endothelial cells by immunofluorescence staining. We showed for the first time that in HUVECs, AJAP1 localized with the microtubule cytoskeleton. This association was persistent and was not altered by cell confluence or duration of culture or in a three-dimensional cell context during spheroid sprouting. Recently, a first hint on this altered localization has been given by demonstrating a cytoplasmic localization of AJAP1 in epithelial cells of the murine mammary gland *in vivo* (Klemmt et al., 2016).

When AJAP1 was present and bound to microtubules, the migratory behavior of cells in two-dimensional cell culture experiments as well as the sprouting behavior in three-dimensional sprouting angiogenesis assays was reduced. On the other hand, when the amount of microtubule-bound AJAP1 was reduced, migration and sprouting were increased. So far, our results suggest that AJAP1 might affect microtubule dynamics during cell migration.

Our results deviate from previous studies. Initially, AJAP1 has been described as a putative transmembrane protein. This has been shown in human mammary gland tissue sections and by overexpression studies in several cell lines. There, it interacts with the E-cadherin-catenin complex (Bharti et al., 2004; Gross et al., 2009). We showed that endogenous AJAP1 interacted with microtubules also in the epithelial cell line MCF-7 and demonstrated that the overexpressed fluorescent fusion protein was not detected by the anti-AJAP1 antibody, which detected the endogenous protein. This may be caused by steric hindrance of the EGFP in the fluorescent fusion protein, which masks the epitope binding site for the antibodies. However, in the glioblastoma context, it has been observed that upon restoration of AJAP1 expression, the cytoskeleton is altered. AJAP1 overexpressing glioblastoma cell lines display a change in the cellular distribution of F-actin and β-tubulin (Han et al., 2014). It has been shown recently that at least three protein isoforms of AJAP1 exist in the human context which show different expression patterns in organs and during developmental processes (Klemmt et al., 2016). This also suggests that the different AJAP1 isoforms can localize differentially within cells. According to the different epitopes of the antibodies used in this study, at least AJAP1 isoform 1 and/or isoform 2 are expressed in HUVECs (Table S1); however, we cannot entirely exclude that isoform 3 is not expressed in HUVECs.

**Fig. 5.**
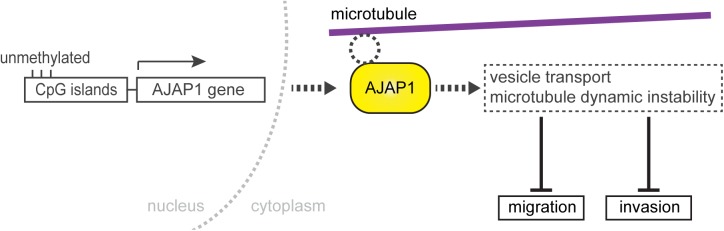
**A hypothetic model showing the role of AJAP1 in primary endothelial cells.** In HUVECs, AJAP1 localizes at the microtubules. It is either a direct binding of AJAP1 or an association via an adaptor protein or protein complex. The interaction with microtubules affects cell migration and invasion, thereby suggesting an attenuating effect on angiogenesis. We hypothesize that these effects are the result of microtubule-bound AJAP1 influencing vesicle transport regulation mechanisms by decreasing trafficking velocity or affecting microtubule dynamic instability by a stabilizing effect. In invasive tumors, it was shown that AJAP1 expression is deregulated by hypermethylation of its promoter. We consider this regulation mechanism of AJAP1 expression in endothelial cells.

Previously, antibodies that target endogenous AJAP1 had been rare. It has been shown in MCF7 cells that endogenous AJAP1 co-localizes with β-catenin (Gross et al., 2009), which we were not able to reproduce. Nevertheless, we provided appropriate controls, i.e. that the fibrillary structures disappeared upon AJAP1 downregulation, showing the specificity of the antibodies. We used state-of-the-art confocal microscopy that provided an improved resolution, and thus obtained subcellular detail. Other studies using cell cultures have focused on the overexpression of a fluorescence protein-tagged AJAP1 to identify its intracellular localization (Gross et al., 2009; Bharti et al., 2004; Jakob et al., 2006; Schreiner et al., 2007), whereas we investigated the localization of the endogenous AJAP1 protein using five available antibodies. These recognize different epitopes on the AJAP1 peptide sequence to get reliable information about its localization within the cell. All AJAP1 antibodies recognized fibrillar structures, although the staining was not restricted to the fibrillar structures. Depending on the antibody, the signal also occurred in puncta, suggesting that it might also be associated with vesicles. The association of AJAP1 with microtubules was not necessary for the maintenance of the cytoskeletal structure. It remains elusive whether the dynamic instability of microtubules is affected by AJAP1. Our findings showed that AJAP1 binds either directly or indirectly via adaptor proteins to the microtubules. To identify potential microtubule binding sites, we searched for similarities between AJAP1 and known microtubule-associated proteins using ScanProsite (de Castro et al., 2006). Based on the *in silico* search we could not identify motifs that were similar to any microtubule-associated protein (Table S2). Therefore, we assume that AJAP1 binds indirectly to the microtubule cytoskeleton, which requires further experiments. We hypothesize that AJAP1 interferes either with the vesicular transport towards the leading edge of the migratory cells by forming a physical barrier for increased vesicular trafficking or that the microtubule-associated AJAP1 affects the dynamic instability of microtubules by stabilization. This decreases the turnover of microtubules, consequently slowing down cell migration (Fig. 5).

In breast cancer cells, AJAP1 has been shown to modulate epithelial growth factor (EGF)-dependent E-cadherin internalization, a process that occurs during tissue remodeling and pathological processes such as tumor development (Gross et al., 2009). Thereby, AJAP1 interferes with the dynamics of adherens junctions, and is involved in the pre-formation of the E-cadherin/EGF receptor (EGFR) HER2/src-kinase/AJAP1 signaling complex. Additionally, overexpression of AJAP1 accelerates E-cadherin internalization. It might be interesting to investigate whether this phenomenon can also be found in the endothelial context or whether it is restricted to epithelial cells. In particular, it raises the questions of whether AJAP1 interacts with growth factor receptors such as the vascular endothelial growth factor receptor 2 (VEGFR2), which was recently shown to localize at microtubules (Czeisler and Mikawa, 2013), and whether AJAP1 expression and localization is altered upon growth factor stimulation.

In conclusion, this study emphasizes that AJAP1 is a putative negative regulator of angiogenesis by reducing cell migration and invasion in endothelial cells. Here we show that AJAP1 has a modulating effect on sprouting angiogenesis and cell migration in HUVECs. We also describe for the first time that AJAP1 localizes to the microtubule cytoskeleton in primary endothelial cells. The degree of this interaction remains elusive, but so far, reduction of AJAP1 expression does not destabilize the microtubules. Thus, we suggest to use the name ‘AJAP1’ with care and re-consider ‘shrew-1’. The acronym AJAP1 implies a restricted localization of the protein to the adherens junctions. However, we show that in endothelial cells, the localization of AJAP1 cannot be assigned to the adherens junction.

Concordant with previous studies, our results suggest AJAP1 as a novel negative feedback regulator of cell migration and invasion, which is ubiquitous and independent from the cellular context. Due to AJAP1s implication in tumor development, progression and vascularization it might serve as a novel diagnostic marker.

## MATERIALS AND METHODS

### Cell culture and transfection

MCF-7 wild-type and MCF-7 AJAP1:EGFP cells were maintained in DMEM (GIBCO) supplemented with 10% FBS (GIBCO) and 2 mM L-Glutamine (GIBCO) at 37°C, and 5% CO_2_. Pre-screened HUVECs (C-12205, Lot: 0061801, 1071101.1, 2012601.1, PromoCell, Heidelberg, Germany) were, if not stated otherwise, maintained in EBM (CC-3121, Lonza, Basel, Switzerland) supplemented with hydrocortisone, bovine brain extract, epidermal growth factor (all from Lonza), and 10% FBS (GIBCO) at 37°C, and 5% CO_2_.

Experiments with HUVECs were approved by: Declaration of Helsinki; German Federal Data Protection Act; Human Tissue Act; and Convention for the protection of Human Rights and Dignity of the Human Being with regard to the Application of Biology and Medicine: Convention on Human Rights and Biomedicine.

12.5 µM small interfering RNAs (siRNAs) (Sigma Aldrich, St. Louis, MO USA) were transfected using peqFECT (Peqlab, Erlangen, Germany) for AJAP1 down-regulation during the spheroid-based sprouting assay, or using Lipofectamine RNAiMAX (Thermo Fisher, Waltham, MA, USA) or Interferin (Peqlab, Erlangen, Germany) in the wound healing assay according to manufacturer's instructions. siAJAP1-1: SASI_Hs01_00244567; siAJAP1-2: SASI_Hs02_00352284; siAJAP1-3: SASI_Hs01_00244565. As a control, a scrRNA (SIC002-10NMOL) was used.

### RNA extraction and quantitative real-time PCR (qRT PCR)

Total RNA was extracted using TRIzol^®^ reagent (15596-026, Life Technologies, Carlsbad, CA, USA,). 1 µg RNA was reverse transcribed using the Maxima First Strand cDNA Synthesis kit (K1642, Thermo Fisher Scientific). The reaction mix was first incubated for 10 min at 25°C, and then for 30 min at 55°C with a subsequent termination for 5 min at 85°C.

Probe-based quantitative real-time PCR was carried out using Taqman Universal PCR Master Mix (4369016, Life Technologies) and Taqman assay probes (AJAP1: Hs00982497_m1; RPS13: Hs01945436_u1, Life Technologies). cDNA was diluted 1:2 with RNase-free water (AM9938, Ambion, Thermo Fisher Scientific). Data was acquired with the CFX-96 detection system (Bio-Rad, Hercules, CA, USA). Experiments were run in triplicate.

### Western blot

HUVECs were lysed in a buffer containing 0.5% sodium deoxycholate, 1% NP-40, 0.1% sodium dodecyl sulfate, 1 mM EDTA in PBS, and freshly added protease inhibitors (Sigma-Aldrich). Proteins were resolved on SDS-polyacrylamide gels, and transferred onto nitrocellulose membranes (GE Healthcare, Little Chalfont, UK). Membranes were blocked with fat-free dry milk in TBS-Tween20. Primary antibodies against GAPDH (AM4300, Ambion), and AJAP1 (ab121361, Abcam, Cambridge, UK) were incubated over night at 4°C. Secondary horseradish peroxidase-conjugated antibodies (115-035-003, 111-035-003, Jackson Immuno Research, Newmarket, UK) were incubated for 1.5 h at room temperature. Protein bands were visualized with an enhanced luminescence detection reagent in the Chemocam documentation system (Intas, Goettingen, Germany). Western blots were analyzed with Image Studio Lite Ver. 5.2 (LI-COR Biosciences, Lincoln, Nebraska, USA).

### Spheroid-based sprouting assay

Spheroid-based sprouting assay was performed as described previously (Diehl et al., 2007). Briefly, 8×10^4^ HUVECs were transfected with siRNA and scrRNA after 24 h, respectively. After 24 h incubation, spheroid formation was performed by hanging-drop technique with 25 µl drops consisting of 400 cells per spheroid and methylcellulose solution (Korff and Augustin, 1998). After one day of formation, spheroids were embedded into collagen gels. Solidified spheroid-containing collagen gels were covered with 100 µl culture medium supplemented with 30 µM bFGF (Invitrogen, Carlsbad, CA, USA). Sprout formation was examined 24 h after embedment by light microscopy (Axiovert, Carl Zeiss, Oberkochen, Germany). For each condition, six experiments were performed with the measurement of ten spheroids per experiment. The cumulative length and the number of sprouts were determined for each spheroid [ImageJ 1.48o (NIH)]. Data analysis was performed with randomized data.

### Immunofluorescence and confocal microscopy

Cells grown on glass coverslips were fixed, permeabilized and blocked with 10% FBS/PBS. The primary antibodies were incubated over night at 4°C. Primary antibodies were α-tubulin [ab52866 (Abcam), A11126 (Life Technologies)] and five different antibodies to detect AJAP1 [ab121361 (Abcam), custom made antibody (Nanotools, Teningen, Germany), clone F (Genovac Aldevron, Freiburg, Germany), AAS47449C, AAS46449C (Antibody verify, Las Vegas, NV, USA)]. Secondary antibodies were Promo Fluor 488 (PK-PF488-AK-M1, PromoKine), Alexa Fluor 488 (A11055, Molecular Probes), and Alexa Fluor 568 (A11011, Molecular Probes). Cell nuclei were counter stained with 1 µg/ml DAPI (Merck, Darmstadt, Germany). Coverslips were mounted with Mowiol and examined with the LSM780 confocal microscope using the Zen software (Carl Zeiss).

For immunofluorescence of sprouted HUVECs, spheroids were embedded in collagen as described above and cultivated in LabTekII (Thermo Fisher Scientific) chambers. The staining protocol was adapted and modified from Wozniak and Keely (2005). Briefly, medium was removed, gels were washed with PBS and fixed with 4% paraformaldehyde. Specimens were incubated with 150 mM glycine and further washed with PBS. Permeabilization was performed with 0.05% Triton X-100, blocking with 10% FBS in 0.01% PBS-T. Spheroids were incubated with the primary antibody in block solution overnight at 4°C, and for 1.5 h with secondary antibody at room temperature. Finally, specimens were mounted with Mowiol and subsequently imaged with the LSM780 confocal microscope using the Zen software (Carl Zeiss).

### Structures illumination microscopy (SIM)

SIM images were acquired using the Elyra PS.1 microscope (Carl Zeiss). We used a Plan-Apochromat 63×/NA 1.4 oil objective and for excitation, a 561 nm and a 488 nm laser used. For affine-alignment we used a multi spec calibration tool (cat. no. 178-455, Carl Zeiss). 1024×1024 pixel images with five different phases for five distinct grid rotations were acquired with a pco.edge sCMOS camera (PCO) and processed with Zen software (Carl Zeiss).

### Wound healing

7.5×10^4^ HUVECs were seeded per 24-well plate and transfected with siRNA after 24 h. One day after transfection, the transfection reagent was removed and growth medium was added for another 24 h. The wound was placed with a 10 µl pipette tip, the culture was washed once with PBS, and incubated with EBM2 (CC-3124, Lonza) in the Axio Observer.Z1 (Carl Zeiss) incubation chamber for 24 h with images acquired every 4 h. Wound areas were measured manually (Image J).

### MTS assay

7.5×10^3^ cells were seeded per 96-well plate and incubated for 24 h. Another 24 h after siRNA transfection, the reagent was removed and growth medium was added for further 24 h. Cells treated with 0.05% Triton X-100 served as a negative control. 25 µl MTS reagent (Promega, Madison, USA) were added to each well with fresh growth medium. The cells were then incubated at culture conditions for 4 h. The absorbance at 570 nm was measured with a microplate reader (Infinite M200, Tecan, Maennedorf, Switzerland). Three independent experiments with six technical replicates were performed.

### Live dead assay

7.5×10^3^ HUVECs were seeded per 96-well plate and incubated for 24 h before treatment with 12.5 µM nocodazole or vehicle (DMSO) and incubated overnight. As a negative control, cells were treated with 0.05% Triton X-100 for 30 min under culture conditions. The cells were then washed and growth medium was supplemented with 2.5 µM SYTOX Orange Nucleic Acid Stain (Thermo Fisher Scientific), for staining of dead cells and 5 µg/ml Hoechst 33342 (Life Technologies) and added to the cells for 20 min at 37°C, 5% CO_2._ Fluorescence intensity of SYTOX Orange Nucleic Acid Stain at was measured: Excitation wavelength: 530 nm, Emission wavelength: 570 nm with a multiplate reader (Infinite M200, Tecan). Hoechst: Excitation wavelength: 361 nm, Emission wavelength: 486 nm. Three independent experiments with three technical replicates were performed.

### Statistical analysis

For the comparison of two independent samples, a two-sample Student's *t*-test was chosen. The results were corrected according to Bonferroni for multiple tests. Data were considered significant at *P*<0.05. Data are presented as means±s.d., or box and whisker plots, where the box contains 50% of the data points, the middle line of the box is the median, and the square is the arithmetic mean. Whiskers show minimum and maximum values.

Data visualization and analysis were performed with OriginPro (version b9.2.257, OriginLab Corporation, Northampton, MA, USA) or Mathematica (version 11, Wolfram Research Inc., Oxfordshire, UK).

### Sequence analysis

Protein domains and functional sites were analyzed using ScanProsite tool (de Castro et al., 2006; http://prosite.expasy.org/prosite.html).
